# Associations of circadian syndrome with gout and hyperuricemia: a cross‑sectional analysis of NHANES 2007–2018

**DOI:** 10.1186/s12889-025-23319-6

**Published:** 2025-06-10

**Authors:** Jun-Wei Ren, Jia-Hao Wang, Yun-Fei Xiao, Yun-Jin Bai, Ping Han

**Affiliations:** 1 Department of Urology, West China Hospital, Sichuan University, Chengdu, China; 2 Institute of Urology, West China Hospital, Sichuan University, Chengdu, China

**Keywords:** Gout, Hyperuricemia, Circadian syndrome, Cross-sectional study, NHANES

## Abstract

**Background:**

Gout and hyperuricemia, driven by elevated uric acid (UA). Circadian syndrome (CircS), a cluster of cardiometabolic risk factors related to circadian disruption, may interact with these conditions, but evidence remains limited. Understanding the potential associations of CircS with gout and hyperuricemia is crucial for effective health interventions.

**Objective:**

To explore the associations of CircS with gout and hyperuricemia.

**Materials and methods:**

This cross-sectional study utilized data from six National Health and Nutrition Examination Survey (NHANES) cycles (2007–2018). Participants aged ≥ 20 years with complete data on gout status, serum UA, and CircS were included. Gout was determined by self-reported physician diagnosis (questionnaire item MCQ160), and hyperuricemia was defined as serum UA > 7.0 mg/dL (men) or > 6.0 mg/dL (women), measured via uricase-based enzymatic assay. Survey-weighted logistic regression models were used to evaluate the associations of CircS with gout and hyperuricemia, adjusting for potential covariates. To assess nonlinear correlations, a restricted cubic spline (RCS) analysis was conducted. Sensitivity analysis was performed to test the stability of results.

**Results:**

The study incorporated a total of 30,157 participants aged 20 years or older, out of which 14,698 were male. After full adjustment, those with CircS had higher odds of gout (OR = 1.34, 95% CI [1.03, 1.73], *P* = 0.03) and hyperuricemia (OR = 1.25, 95% CI [1.11, 1.41], *P* < 0.001) compared with individuals without CircS. A gradual increase in serum UA levels was associated with CircS. The RCS curve displays a nonlinear relationship between UA levels and CircS. Sensitivity analyses, including stratified analyses across key covariates and trend tests for CircS scores, consistently demonstrated that the associations of CircS with gout and hyperuricemia remained robust.

**Conclusion:**

This study identified that CircS was significantly associated with both gout and hyperuricemia in the US general population. The risk of these conditions increased in parallel with rising CircS scores. Elevated UA levels were also associated with a higher incidence of CircS. Given the limitations inherent to the cross-sectional design, further longitudinal studies are required to establish causality and elucidate underlying mechanisms.

**Supplementary Information:**

The online version contains supplementary material available at 10.1186/s12889-025-23319-6.

## Introduction

Circadian syndrome (CircS) is characterized by a combination of metabolic syndrome (MetS) criteria and two additional circadian-related symptoms. MetS, as a core component, includes at least three of the following: central obesity (elevated waist circumference), elevated blood pressure, reduced high-density lipoprotein cholesterol (HDL-C), elevated triglycerides, and impaired fasting glucose [1]. CircS extends this framework by requiring the presence of two further conditions linked to circadian dysregulation, specifically depressive symptoms and sleep disturbances. This integrative definition captures the interplay of metabolic, emotional, and sleep-related factors associated with circadian dysfunction. Contemporary lifestyle factors like insufficient sleep, high-calorie diets, shift work, and exposure to artificial light disrupt circadian rhythms, leading to diverse health implications [2–6]. CircS is now recognized as a significant underlying factor contributing to MetS and its associated health issues [7]. Given the role of circadian rhythms in metabolic regulation, it is plausible that disruptions in these rhythms may also contribute to conditions such as gout and hyperuricemia. Gout is a multifaceted type of arthritis marked by the build-up of urate crystals in the joints. Acute flare-ups present with inflammatory signs and symptoms, including tenderness, redness, and severe pain, most commonly affecting the big toe’s metatarsophalangeal joint. However, it can also impact other joints such as the foot, ankle, knee, wrist, and elbow, often striking at night [8]. Hyperuricemia is defined as an elevated level of UA in the blood, which can be associated with various health conditions, including gout, Mets and cardiovascular diseases [9, 10]. Persistent hyperuricemia can lead to the deposition of monosodium urate (MSU) crystals in joint structures, ultimately triggering an innate immune response to the deposited crystals mediated by the NOD-like receptor protein 3 (NLRP3) inflammasome [11]. An increasing body of research indicates that CircS elevates risks of certain conditions, such as cardiovascular diseases [2], frailty [12], overactive bladder [13], and gallstone [14]. However, its potential association with gout and hyperuricemia remains underexplored. This study investigates the associations of CircS with gout and hyperuricemia in the US population, aiming to provide data-supported evidence to clarify these conditions.

## Materials and methods

### Data sources and ethics

The National Health and Nutrition Examination Survey (NHANES), conducted by the Centers for Disease Control and Prevention (CDC), is a recurring national survey managed by the National Center for Health Statistics (NCHS). It systematically evaluates the health and nutritional status of the American population every two years, aiming to gather comprehensive data on contemporary disease trends to inform public health policies. The NHANES data is publicly accessible and can be freely downloaded from: https://www.cdc.gov/nchs/nhanes/index.htm, https://wwwn.cdc.gov/nchs/nhanes/. Also, this data has been de-identified to protect participant privacy, requiring no further approval and adhering to ethical guidelines.

### Data collection and definition

**Fig. 1:**
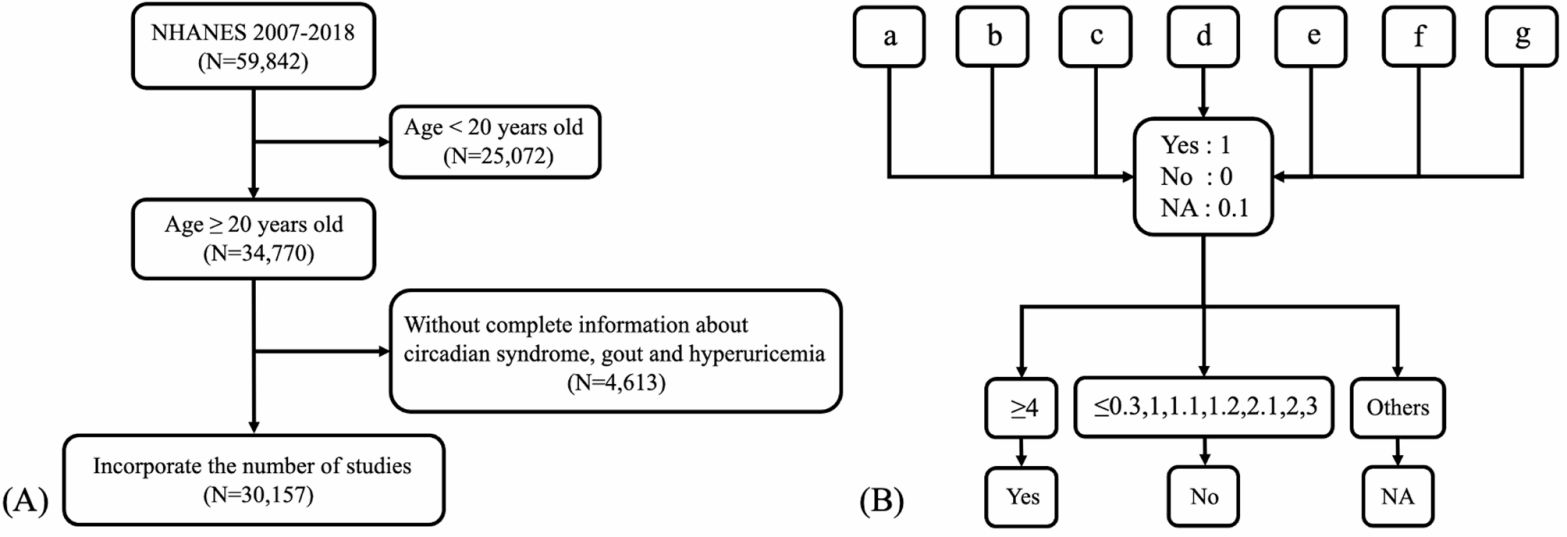
Flowchart of final inclusion population and defining circadian syndrome. (**A**) Flowchart of final inclusion population. (**B**) Flowchart of defining circadian syndrome. **a**: A waist circumference of at least 88 cm for women and 102 cm for men. **b**: High triglyceride levels (≥ 150 mg/dL) or treatment with lipid-lowering medications. **c**: Low HDL-C (< 40 mg/dL for men and 50 mg/dL for women) or the use of lipid-lowering drugs. **d**: High blood pressure (systolic ≥ 130 mmHg or diastolic ≥ 85 mmHg) or the use of antihypertensive medications. **e**: Elevated fasting blood sugar (≥ 100 mg/dL) or use of diabetes medications. **f**: Short sleep duration (< 6 h/day). **g**: Depressive symptoms assessed using the Patient Health Questionnaire-9 (PHQ-9)

This study utilized NHANES data from six cycles spanning 2007 to 2018 (2007–2008, 2009–2010, 2011–2012, 2013–2014, 2015–2016, and 2017–2018), involving 59,842 individuals. After excluding those under 20 years old (*n* = 25,072) and individuals with incomplete information in the CircS, gout, and hyperuricemia groups (*n* = 4,613), the remaining population comprised 30,157 individuals (Fig. 1A). All survey data includes non-zero weighting information. Demographic ​​variables​​ ​​were​​ extracted from the questionnaire. UA is extracted from laboratory tests and was enzymatically oxidized by uricase to produce hydrogen peroxide, which reacted with 4-aminophenazone (peroxidase-catalyzed) to generate a quantifiable chromophore. Hyperuricemia is defined as a serum UA concentration of 7.0 mg/dL or higher in males, and 6.0 mg/dL or higher in females [15, 16]. Gout status was determined through self-reported physician diagnosis (NHANES questionnaire item MCQ160: “Has a doctor or other health professional ever told you that you had gout?”). Participants who clearly answered “yes” were classified as having gout. As described in previous studies, the presence of four or more of the following elements is diagnostic of CircS [2, 17–19]: (1) Waist circumference ≥ 88 cm for women and ≥ 102 cm for men; (2) Elevated triglycerides (≥ 150 mg/dL) or use of lipid-lowering drugs; (3) Reduced HDL-C (< 40 mg/dL for men, < 50 mg/dL for women) or use of lipid-lowering drugs; (4) Elevated blood pressure (systolic blood pressure ≥ 130 or diastolic blood pressure ≥ 85mmHg) or use of antihypertensive drugs; (5) elevated fasting blood glucose (≥ 100 mg/dL) or use of antidiabetic drugs; (6) Shortened sleep time (< 6 h/day); (7) Depressive symptoms, as measured by the Patient Health Questionnaire-9 (PHQ-9). The relevant variables required to assess the seven CircS diagnostic components were extracted from the database. For each component, a score of 1 was assigned if the diagnostic criterion was met, 0 if the criterion was not met, and 0.1 if the information was missing or could not be evaluated. A total score ≥ 4 was defined as the threshold for CircS. Subsequently, the total score for each participant was calculated to determine individuals who could be classified as having CircS. Participants were categorized into three groups: (1) CircS if the total score was ≥ 4; (2) Non-CircS if the score was ≤ 0.3, or exactly 1, 1.1, 1.2, 2, 2.1, or 3; and (3) Unassessable if the score fell into any other category (Fig. 1B).

### Covariates’ collection

Covariate information was collected through questionnaires, examinations, and laboratory tests. Demographic and behavioral variables, including age, gender, race, education, marital status, poverty income ratio (PIR), smoking status, alcohol consumption, vigorous activity, moderate activity, were obtained through validated questionnaires. Self-reported medical histories, such as cancer and stroke, were also collected via questionnaires. Physical examinations provided measurements for body mass index (BMI). In addition, cardiovascular disease (CVD) was obtained by integrating coronary heart disease, congestive heart failure, heart attack, stroke, and angina in the questionnaire. Hypertension was defined as either a self-reported physician diagnosis or the use of antihypertensive medications based on questionnaires, as well as systolic/diastolic blood pressure ≥ 140/90 mmHg measured in physical examinations. Diabetes mellitus (DM) was defined as either a self-reported physician diagnosis or the use of antidiabetic medications based on questionnaires, as well as meeting the diagnostic criteria through laboratory tests, including glycated hemoglobin, fasting plasma glucose, random blood glucose, or 2-hour plasma glucose after oral glucose tolerance test. Laboratory tests also included measurements of serum creatinine to calculate the estimated glomerular filtration rate (eGFR), with chronic kidney disease (CKD) defined according to the 2021 Kidney Disease: Improving Global Outcomes (KDIGO) guidelines [20]. Continuous variables include age (≥ 20 years), PIR, BMI, and UA. Categorical variables are listed as gender (female, male), race (Mexican American, other Hispanic, non-Hispanic White, non-Hispanic Black, and other races), education (less than 9th grade, 9-11th grade, high school graduate, some college, college graduate or above), marital status (married, widowed, divorced, separated, never married, and living with partner), smoking status (never, former, now), PHQ-9 ([0,9], [10, 27], missing), DM (no, pre-diabetes, diabetes), alcohol consumption (never, former, mild, moderate, heavy, missing), cancer, stroke, CVD, CKD, hypertension, moderate activity, vigorous activity, and short sleep (all categorized as no or yes). Covariables with more than 3% missing data included PIR, PHQ-9, and alcohol consumption. No covariate had a missing rate exceeding 10%. Their missing values were categorized as “Missing” for analysis. Furthermore, continuous variables were converted into categorical variables for statistical comparison. Age was categorized into four groups (20–34, 35–49, 50–64, and ≥ 65 years) to balance statistical precision and age-specificity. BMI was grouped into three categories (< 25, 25–30, and ≥ 30 kg/m²) and PIR was divided into three categories (≤ 1.3, 1.3–3.5,>3.5) with missing values as a separate category, to consistent with prior studies [13, 21].

### Statistical methods

We used the sampling weights, stratification, and clustering provided by the NHANES study to account for the complex, multistage sampling design aimed at selecting a representative sample of the noninstitutionalized U.S. population. Continuous variables are presented as weighted survey means and standard error (SE), while categorical variables are depicted as sample and weighted survey proportions. For assessing the associations of CircS with gout and hyperuricemia, we utilized logistic regression analysis and further multivariable regression models. Otherwise, the RCS curve was used to explore the nonlinear relationship between UA and CircS. Multiple subgroup analyses showed that the conclusion was stable. R software version 4.4.1 was used for all statistical analyses, and when a two-tailed P-value < 0.05, the differences were statistically significant.

## Results

### Baseline characteristics of the population

A total of 59,842 respondents’ information was extracted from the database. First, individuals under 20 years of age were excluded (*n* = 25,072), as the survey only involved adults. Further exclusions were made for participants with missing data on gout (*n* = 41), hyperuricemia (*n* = 3,501), and CircS (*n* = 1,811), leaving a total of 30,157 participants with a weighted mean age (SE) of 47.44 (0.23) years, comprising 48.43% males and 51.57% females. The weighted mean UA (SE) of the included population was 5.41 (0.01) mg/dL. The results revealed statistically significant differences between CircS and non-CircS populations across all variables except gender (Table 1). The non-CircS group included 20,773 participants, whereas the CircS group comprised 9,384 participants. Compared to the non-CircS group, individuals with CircS were older (mean ± SE, 57.75 ± 0.23 years), had higher UA levels (mean ± SE, 5.85 ± 0.02 mg/dL), and a higher BMI (mean ± SE, 32.77 ± 0.12 kg/m²). Multivariable logistic regression analysis revealed significant associations between CircS and multiple risk factors, including older age, higher BMI, marital status (widowed or divorced compared with married), smoking, cancer, stroke, CKD, CVD, gout, and hyperuricemia (Figure S1). Additionally, the age distribution in the CircS group was predominantly 50 years and older, with a notable increase in the number of individuals with gout and hyperuricemia in this age range (Table S1).

In the study of patients with or without gout, all variables except PIR, education, PHQ-9, vigorous activity, and moderate activity showed statistically significant differences. Similarly, in the analysis of patients with or without hyperuricemia, all variables except PIR, vigorous activity, moderate activity, and short sleep demonstrated statistically significant differences. Compared to the non-gout group, individuals with gout were older, and likewise, those with hyperuricemia were older compared to the non-hyperuricemia group. Regarding gender distribution, among females (*n* = 15,459), 2.43% had gout and 16.44% had hyperuricemia. Among males (*n* = 14,698), the prevalence of gout and hyperuricemia was higher, at 5.66% and 22.44%, respectively (Table S1). According to the diagnostic criteria for CircS, each fulfilled criterion contributed 1 point, yielding a total score (range: 0–7) for each participant. A score ≥ 4 defined CircS, while scores < 4 indicated non-CircS. Notably, among those with CircS (score ≥ 4), the majority (over 50%) had a score of exactly 4. Moreover, the prevalence of gout and hyperuricemia increased progressively with higher CircS scores.

**Fig. 2:**
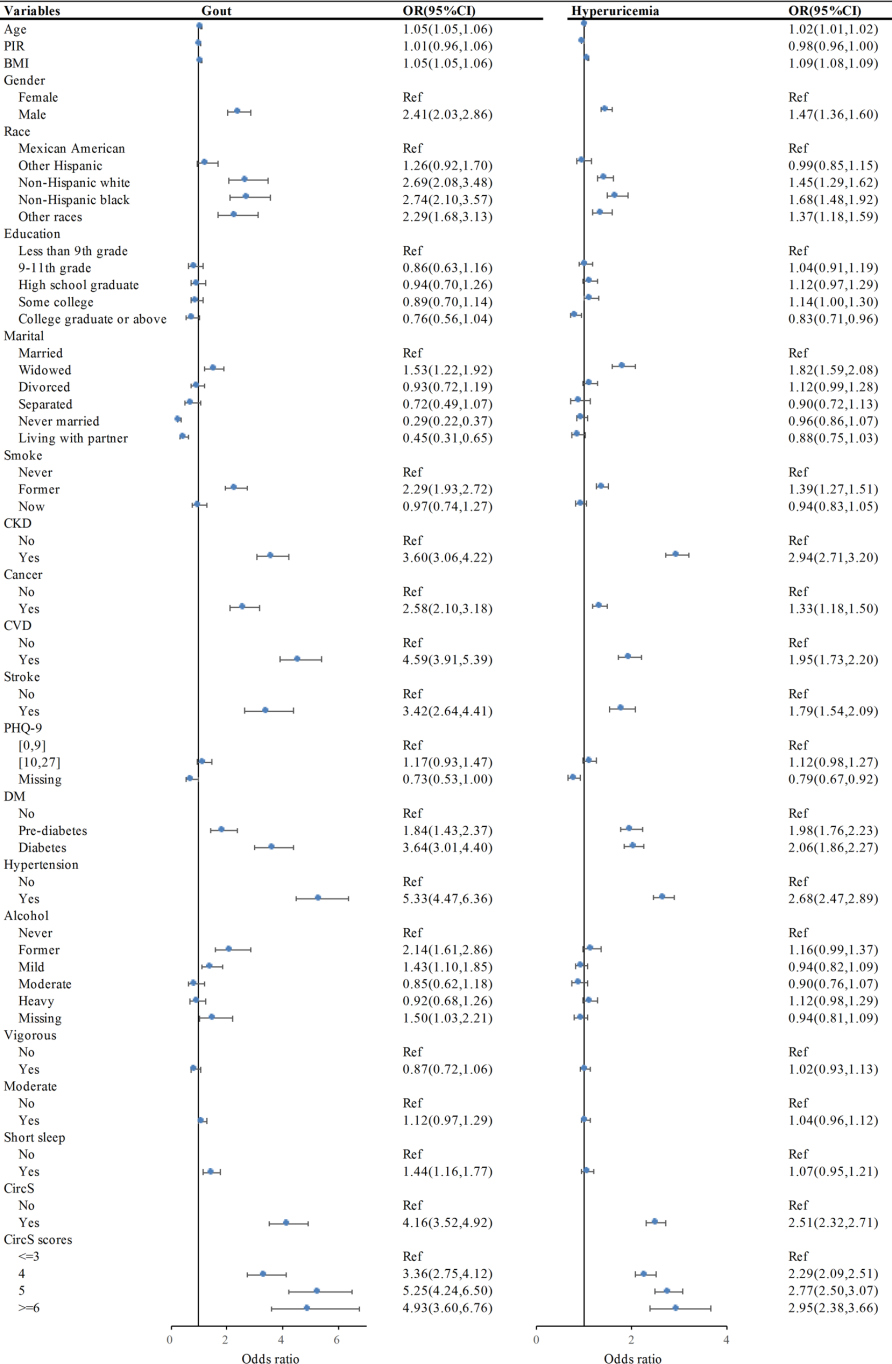
Logistic regression analysis of variables associated with gout and hyperuricemia. CI, confidence interval; OR, odds ratio; UA, uric acid; PIR, poverty income ratio; BMI, body mass index; CKD, chronic kidney disease; CVD, cardiovascular disease; PHQ-9, Patient Health Questionnaire-9; DM, diabetes mellitus; CircS, circadian syndrome

Furthermore, to explore the independent effects of these characteristics on gout and hyperuricemia, logistic regression analyses of the variables associated with both conditions are visualized (Fig. 2). A reference category was used for each categorical variable. Gout and hyperuricemia exhibited overlapping risk determinants spanning three interconnected biological domains: metabolic dysregulation (elevated BMI, DM, and hypertension), end-organ damage (CKD, CVD, and stroke), and circadian disruption (as represented by CircS).

**Table 1:** Characteristics of participants by categories of circadian syndrome: NHANES 2007–2018

		Circadian Syndrome		
Variables	Total (30,157)	No (20,773)	Yes (9,384)	P value
UA	5.41 (0.01)	5.25 (0.02)	5.85 (0.02)	< 0.0001
Age	47.44 (0.23)	43.53 (0.25)	57.75 (0.23)	< 0.0001
20–34	7,527 (27.49)	6,913 (35.01)	614 (7.60)	
35–49	7,594 (27.58)	5,959 (30.18)	1,635 (20.73)	
50–64	7,929 (26.61)	4,735 (22.90)	3,194 (36.40)	
>=65	7,107 (18.32)	3,166 (11.90)	3,941 (35.27)	
Gender				0.55
Female	15,459 (51.57)	10,549 (51.69)	4,910 (51.22)	
Male	14,698 (48.43)	10,224 (48.31)	4,474 (48.78)	
PIR	3.00 (0.04)	3.06 (0.04)	2.86 (0.04)	< 0.0001
<=1.3	8,848 (20.15)	5,892 (19.64)	2,956 (21.52)	
1.3–3.5	10,272 (32.78)	6,940 (31.73)	3,332 (35.55)	
>3.5	8,249 (39.58)	6,044 (41.20)	2,205 (35.32)	
Missing	2,788 (7.49)	1,897 (7.44)	891 (7.61)	
BMI	29.04 (0.09)	27.64 (0.08)	32.77 (0.12)	< 0.0001
<25	8,584 (29.37)	7,720 (37.56)	864 (8.37)	
25–30	9,846 (32.82)	7,053 (34.55)	2,793 (29.06)	
>=30	11,450 (37.12)	5,847 (27.89)	5,603 (62.57)	
Race				< 0.0001
Mexican American	4,604 (8.61)	3,197 (9.05)	1,407 (7.46)	
Other Hispanic	3,190 (5.90)	2,166 (6.19)	1,024 (5.11)	
Non-Hispanic white	12,480 (66.77)	8,260 (65.32)	4,220 (70.63)	
Non-Hispanic black	6,195 (10.65)	4,318 (10.86)	1,877 (10.10)	
Other races	3,688 (8.07)	2,832 (8.58)	856 (6.71)	
Education				< 0.0001
Less than 9th grade	3,114 (5.25)	1,760 (4.40)	1,354 (7.50)	
9-11th grade	4,202 (10.35)	2,734 (9.62)	1,468 (12.31)	
High school graduate	6,866 (22.88)	4,589 (21.75)	2,277 (25.92)	
Some college	8,934 (31.58)	6,226 (31.29)	2,708 (32.42)	
College graduate or above	7,009 (29.88)	5,446 (32.95)	1,563 (21.85)	
Marital				< 0.0001
Married	15,525 (55.39)	10,448 (54.32)	5,077 (58.29)	
Widowed	2,326 (5.59)	1,066 (3.67)	1,260 (10.68)	
Divorced	3,321 (10.26)	2,020 (9.04)	1,301 (13.49)	
Separated	1,020 (2.38)	689 (2.34)	331 (2.48)	
Never married	5,501 (18.16)	4,573 (21.49)	928 (9.38)	
Living with partner	2,450 (8.19)	1,970 (9.14)	480 (5.68)	
Smoke				< 0.0001
Never	16,822 (55.69)	12,216 (58.56)	4,606 (48.17)	
Former	7,220 (24.71)	4,223 (21.55)	2,997 (33.11)	
Now	6,099 (19.56)	4,322 (19.89)	1,777 (18.72)	
CKD				< 0.0001
No	24,546 (85.20)	18,261 (90.57)	6,285 (72.99)	
Yes	5,382 (14.16)	2,364 (9.43)	3,018 (27.01)	
Cancer				< 0.0001
No	27,309 (89.85)	19,317 (92.40)	7,992 (83.38)	
Yes	2,825 (10.06)	1,444 (7.60)	1,381 (16.62)	
CVD				< 0.0001
No	26,869 (91.38)	19,745 (96.17)	7,124 (78.74)	
Yes	3,286 (8.62)	1,026 (3.83)	2,260 (21.26)	
Stroke				< 0.0001
No	28,950 (97.03)	20,354 (98.54)	8,596 (93.37)	
Yes	1,175 (2.87)	403 (1.46)	772 (6.63)	
PHQ-9				< 0.0001
[0,9]	25,589 (86.85)	18,330 (89.49)	7,259 (79.89)	
[10,27]	2,592 (7.56)	1,053 (4.71)	1,539 (15.10)	
Missing	1,976 (5.58)	1,390 (5.80)	586 (5.01)	
DM				< 0.0001
No	21,441 (75.99)	17,561 (88.58)	3,880 (46.15)	
Pre-diabetes	2,545 (8.43)	1,379 (6.29)	1,166 (14.33)	
Diabetes	5,856 (14.50)	1,532 (5.13)	4,324 (39.52)	
Hypertension				< 0.0001
No	17,372 (62.50)	14,921 (75.43)	2,451 (28.34)	
Yes	12,784 (37.50)	5,851 (24.57)	6,933 (71.66)	
Alcohol				< 0.0001
Never	3,937 (10.01)	2,546 (9.46)	1,391 (11.46)	
Former	4,277 (11.65)	2,358 (9.33)	1,919 (17.77)	
Mild	9,242 (33.91)	6,396 (33.31)	2,846 (35.51)	
Moderate	4,257 (16.26)	3,272 (17.77)	985 (12.25)	
Heavy	5,592 (19.89)	4,328 (22.11)	1,264 (14.02)	
Missing	2,852 (8.29)	1,873 (8.02)	979 (9.00)	
Vigorous				< 0.0001
No	24,199 (78.05)	16,344 (76.88)	7,855 (81.17)	
Yes	5,951 (21.94)	4,424 (23.12)	1,527 (18.83)	
Moderate				< 0.0001
No	19,038 (58.27)	12,763 (57.15)	6,275 (61.26)	
Yes	11,106 (41.71)	8,002 (42.85)	3,104 (38.74)	
Short Sleep				< 0.0001
No	26,008 (88.46)	18,773 (92.29)	7,235 (79.01)	
Yes	4,074 (11.32)	1,963 (7.71)	2,111 (20.99)	
Gout				< 0.0001
No	28,748 (96.01)	20,246 (97.77)	8,502 (91.34)	
Yes	1,409 (3.99)	527 (2.23)	882 (8.66)	
Hyperuricemia				< 0.0001
No	23,988 (80.65)	17,590 (84.96)	6,398 (69.26)	
Yes	6,169 (19.35)	3,183 (15.04)	2,986 (30.74)	

Data are presented as survey-weighted mean (stand error) for continuous variables and sample (survey-weighted percentage) for categorical variables. The t-test for slope was used in survey-weighted generalized linear models. UA, uric acid; PIR, poverty income ratio; BMI, body mass index; CKD, chronic kidney disease; CVD, cardiovascular disease; PHQ-9, Patient Health Questionnaire-9; DM, diabetes mellitus

### Multivariable regression analysis

Univariable followed by multivariable logistic regression was conducted to investigate the associations of CircS with gout and hyperuricemia. The multivariable analysis revealed a positive association of gout prevalence with CircS compared to those without CircS in the crude model (OR = 4.16, 95% CI [3.52, 4.92], *P* < 0.0001), the minimally adjusted model (OR = 1.33, 95% CI [1.04, 1.71], *P* = 0.02), and the fully adjusted model (OR = 1.34, 95% CI [1.03, 1.73], *P* = 0.03). Similarly, a positive association of hyperuricemia prevalence with CircS was observed compared to those without CircS in the crude model (OR = 2.51, 95% CI [2.32, 2.71], *P* < 0.0001), the minimally adjusted model (OR = 1.21, 95% CI [1.10, 1.34], *P* < 0.001), and the fully adjusted model (OR = 1.25, 95% CI [1.11, 1.41], *P* < 0.001) (Table 2).

**Table 2:** Association of circadian syndrome with both gout and hyperuricemia

	Crude model		Model 1		Model 2	
	OR (95%CI)	P value	OR (95%CI)	P value	OR (95%CI)	P value
Gout						
No	Ref		Ref		Ref	
Yes	4.16 (3.52,4.92)	**< 0.0001**	1.33(1.04,1.71)	**0.02**	1.34(1.03,1.73)	**0.03**
Hyperuricemia						
No	Ref		Ref		Ref	
Yes	2.51 (2.32,2.71)	**< 0.0001**	1.21(1.10,1.34)	**< 0.001**	1.25(1.11,1.41)	**< 0.001**

Crude model: Circadian syndrome

Model 1 for gout: Circadian syndrome, Age, BMI, Gender, Cancer, CVD, Stroke, CKD, DM, Hypertension, Short sleep

Model 1 for Hyperuricemia: Circadian syndrome, Age, BMI, Gender, Cancer, CVD, Stroke, CKD, DM, Hypertension

Model 2: Circadian syndrome, Age, PIR, BMI, Gender, Race, Education, Marital, Smoke, Cancer, CVD, CKD, Stroke, PHQ-9, DM, Hypertension, Alcohol, Vigorous, Moderate, Short sleep

CI, confidence interval; OR, odds ratio; PIR, poverty income ratio; BMI, body mass index; CKD, chronic kidney disease; CVD, cardiovascular disease; PHQ-9, Patient Health Questionnaire-9; DM, diabetes mellitus

### CircS and serum UA levels

**Fig. 3:**
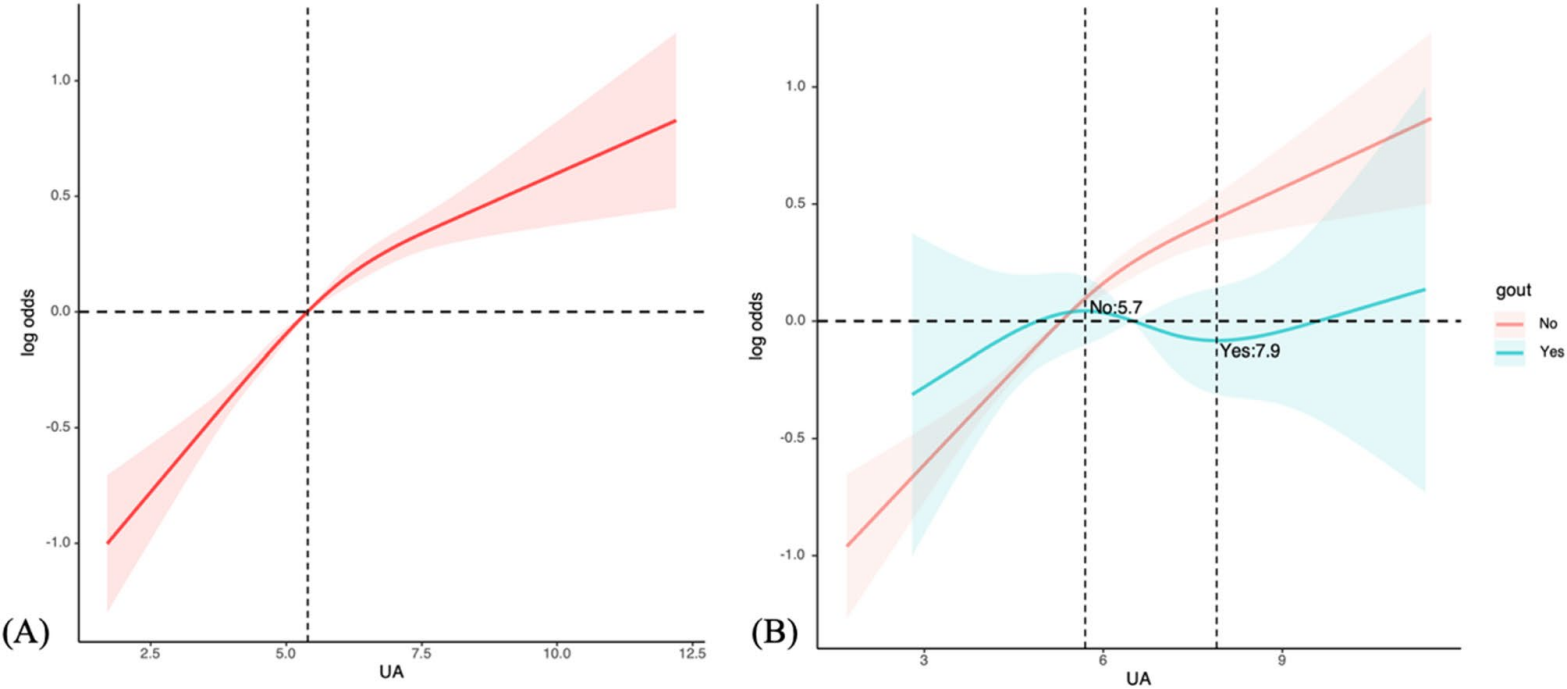
RCS analysis of the nonlinearity between UA and CircS. (**A**) The RCS curve of the association between UA and CircS among all the study participants. RCS regression was adjusted for age, PIR, education, smoke, cancer, CVD, stroke, CKD, DM, hypertension, vigorous activity, moderate activity, short sleep. P value for nonlinear: 0.0034. (**B**) The RCS curves of the association between UA and CircS among non-gout (red curve) and gout (blue curve) respectively. RCS regression was adjusted for age, PIR, education, smoke, cancer, CVD, stroke, CKD, DM, hypertension, vigorous activity, moderate activity, short sleep. P value for nonlinear in non-gout group: 0.0177; P value for nonlinear in gout group: 0.6176. RCS, restricted cubic spline; UA, uric acid; PIR, poverty income ratio; CKD, chronic kidney disease; CVD, cardiovascular disease; DM, diabetes mellitus

UA is a risk factor for gout and determines whether hyperuricemia exists. Thus, we used RCS regression with four knots to assess the nonlinearity between UA and CircS in all participants. When grouped by gout status, the nonlinear relationship between UA and CircS persisted in the non-gout group, whereas the RCS model failed to capture a similar relationship in the gout group (Fig. 3). Furthermore, the weighted characteristics were divided into quartiles based on UA levels: Q1 (0.4–4.4 mg/dL), Q2 (4.4–5.3 mg/dL), Q3 (5.3–6.4 mg/dL), and Q4 (6.4–18 mg/dL). When participants were categorized by the quartile of UA levels, there was a positive association between the incidence of CircS and UA levels by a trend test analysis, and P value was less than 0.001 in all the three models (Table 3).

**Table 3:** Association between the incidence of circs and UA levels

UA levels	*N* (Per)	Crude model		Model 1		Model 2	
		OR (95%CI)	P value	OR (95%CI)	P value	OR (95%CI)	P value
Q1 [0.4,4.4]	26.09%	Ref		Ref		Ref	
Q2 (4.4,5.3]	24.21%	1.52(1.37,1.69)	**< 0.0001**	1.14(0.99, 1.31)	0.07	1.22(1.05, 1.41)	**0.01**
Q3 (5.3,6.4]	26.42%	2.12(1.90,2.37)	**< 0.0001**	1.28(1.11, 1.48)	**< 0.001**	1.46(1.26, 1.70)	**< 0.0001**
Q4 (6.4,18]	23.29%	3.07(2.77,3.40)	**< 0.0001**	1.33(1.16, 1.52)	**< 0.0001**	1.64(1.41, 1.91)	**< 0.0001**
P Value for Trend			**< 0.0001**		**< 0.0001**		**< 0.0001**

Crude model: UA

Model 1: UA, Age, BMI, Education, Smoke, Cancer, CVD, Stroke, CKD, DM, Hypertension, Vigorous, Moderate, Short Sleep

Model 2: UA, Age, PIR, BMI, Education, Gender, Race, Marital, Smoke, Cancer, CVD, Stroke, PHQ-9, DM, Hypertension, Alcohol, Vigorous, Moderate, Short Sleep

CI, confidence interval; OR, odds ratio; UA, uric acid; PIR, poverty income ratio; BMI, body mass index; CKD, chronic kidney disease; CVD, cardiovascular disease; PHQ-9, Patient Health Questionnaire-9; DM, diabetes mellitus

### Sensitivity analyses

To control for confounding and assess whether certain factors may have influenced the associations of CircS with gout and hyperuricemia, a stratified analysis was performed (Table S2). As the results showed, no factor could modify the significant positive associations of CircS with gout and hyperuricemia. In other words, the statistically significant relationship remained robust and was not affected by these covariates. Furthermore, to further explore the progressive relationship in CircS scores, a multivariable regression analysis and trend text was performed in both gout and hyperuricemia (Table S3). The results suggest that there were positive associations of CircS scores with gout and hyperuricemia, as shown by a trend test analysis, with a significant trend observed across all six models. This implies that the risk of gout and hyperuricemia may progressively rise as the CircS score increases, further verifying the findings.

## Discussion

This cross-sectional study aimed to assess the associations of CircS with gout and hyperuricemia. Data were extracted from six cycles of NHANES, spanning 2007 to 2018, and subsequently analyzed. The analysis revealed significant differences between CircS and non-CircS populations, particularly among certain demographic and clinical groups. Furthermore, CircS was associated with multiple risk factors, including metabolic derangements (elevated BMI, UA or fasting glucose), lifestyle factors (smoking), social determinants (marital status), and comorbidities (CVD, CKD and cancer), suggesting that circadian dysregulation may result from synergistic interactions among systemic inflammation, behavioral patterns, and socioeconomic stressors. Additionally, logistic regression analysis showed positive associations of CircS with gout and hyperuricemia. Further stratified analyses confirmed the robustness of the results across various confounding factors. After quantifying CircS as scores, the trend test indicated that an increase in scores might be linearly associated with a higher risk of gout and hyperuricemia. In line with previous studies, this finding reinforces the association between chronodisruption and the occurrence and progression of tumors [22–26]. These pieces of evidence indicate that factors affecting circadian rhythm are ubiquitous in daily life, and identifying their impacts may facilitate primary prevention of diseases, particularly those strongly associated with circadian imbalance.

To further interpret the primary findings, detailed association analyses were conducted using univariable and multivariable logistic regression models. The variables that showed significant differences in the univariable analysis of gout or hyperuricemia were included in a minimally adjusted model, and further incorporated into a fully adjusted model with all covariates. All six models demonstrated statistically significant associations, supporting the robustness of the findings. Subsequently, the RCS curve displayed a nonlinear and positive association between UA and CircS in the overall population. In the non-gout group, the nonlinear relationship remained and was similar to the results from the overall population analysis. However, in the gout group, the RCS curve exhibited a non-monotonic pattern with no statistically significant differences. This suggests that the nonlinear association observed in the overall population is primarily driven by the non-gout individuals, while in gout patients, the relationship between UA and CircS may be more complex or obscured by the influence of gout itself. In non-gout individuals, changes in UA levels may directly affect CircS, whereas in gout patients, the long-standing hyperuricemia and inflammatory state may alter the pattern of UA’s impact on CircS, though this remains to be confirmed [27]. This suggests that when interpreting the effect of UA on CircS, the patient’s gout status must be considered. In the non-gout population, changes in UA levels may require closer monitoring due to the consistently positive nonlinear relationship, while in gout patients, additional factors may need to be considered to assess the risk of CircS. However, the disappearance of this nonlinear relationship may be attributed to several factors. First, the presence of other noise may play a role, as the UA levels between the two change points are close to those of healthy individuals. Second, while change points were observed on the curve, the fluctuations may not be sufficient to establish a strong nonlinear effect. Finally, the smaller sample size in the gout group compared to the full cohort might have impacted the statistical power. Moreover, the CircS scores represent a simple quantification of CircS, developed based on the diagnostic criteria of MetS. Another method for quantifying circadian rhythm is the circadian rhythm score (CRscore), a reliable predictive biomarker for Alzheimer’s disease, closely associated with various immune characteristics, intercellular communication, metabolic pathways, and transcriptional features [28]. In the future, more diverse evaluation methods are needed to enhance the predictive value of CircS for diseases, particularly in developing more objective assessment systems, potentially even pathologically based frameworks.

The circadian rhythm system is a key regulator of nearly all aspects of human health and metabolism. The human brain contains a master “biological clock”, located in the suprachiasmatic nucleus (SCN) of the hypothalamus, which serves as the central conductor of circadian rhythms [29]. This master clock regulates the body’s metabolism by controlling various bodily functions and synchronizing the biological clocks of peripheral organs. It governs a range of physiological processes, including immune function, body temperature, blood pressure, and appetite [3]. Recent research has discovered that the primary cilia of neurons in the brain’s SCN are organelles that regulate the body’s circadian rhythms, revealing the existence of a tangible biological clock and its rhythm-regulating mechanisms [30]. The circadian clock system is organized as a hierarchical network consisting of central and peripheral clocks, responsible for generating, maintaining, and synchronizing daily rhythms [31]. This system includes four main components: a central biochemical clock, input pathways that synchronize the central clock, diverse output pathways, such as hormones and the autonomic nervous system, and peripheral molecular clocks, which regulate tissue-specific transcriptomic expression and physiology [32, 33]. These interconnected elements allow the circadian system to harmonize various physiological processes with environmental changes, such as light and temperature, and ensure homeostasis across organ systems [34].

Additionally, MetS, a cluster of cardiometabolic risk factors and comorbidities, significantly elevates the risk of cardiovascular disease and type 2 diabetes, imposing substantial socioeconomic burdens globally due to its associated morbidity and mortality [35]. Chronic disruption of circadian rhythms is associated with MetS and diseases driven by metabolic dysfunction [36, 37]. Gout patients frequently exhibit multiple comorbidities, including CVD, hypertension, CKD, obesity, MetS, and DM, of which hyperuricemia is a recognized predictor [10, 38]. Emerging evidence links these cardiometabolic risk factors and comorbidities to circadian rhythm disturbances, suggesting a shared etiology for many of these clustered components [2, 39–43]. This implies that circadian dysregulation may play a central role in driving these common risk factors and diseases. Therefore, it is reasonable to propose that circadian rhythm disruption underpins the development of this syndrome, warranting the collective recognition and naming of these conditions as CircS, which includes the components of MetS along with short sleep and depression [3, 44]. Notably, CircS may have higher predictive value than Mets in certain diseases [2, 45, 46]. The introduction of CircS not only expands the conceptual framework of MetS but also advances toward a more robust etiological foundation. It positions circadian disruption as a central mechanism potentially driving the cluster of metabolic risk factors and diseases. This emphasizes the critical role that maintaining a healthy circadian rhythm plays in the prevention and management of MetS, highlighting the importance of circadian regulation in overall metabolic health.

Mechanistically, studies have shown that dysregulation of the signaling cascade is associated with gout, while the circadian clock can regulate the expression and activation of NLRP3, thereby controlling the secretion of IL-1β and IL-18 in various tissues and immune cells, particularly macrophages [47–49]. In the pathogenesis of gout, MSU crystals alter the expression of circadian clock components in macrophages, leading to a loss of inhibition of inflammasome activity. This disruption results in time-of-day-dependent variations in susceptibility to inflammasome activation. The increased incidence of nocturnal gout attacks may be a consequence of altered circadian control of immune cell function [50]. Circadian dysregulation can affect the evolution of autoimmune diseases [27]. The importance of chronobiology in rheumatoid arthritis has been well established, and chronotherapy is widely accepted in the field of rheumatology [51]. This highlights the close link between such diseases and circadian rhythms or chronotherapy. Consistent with our findings, individuals with circadian misalignment are at a higher risk of developing gout, suggesting a potential shared mechanism between circadian rhythm disturbances and gout onset, possibly mediated by immune cells and inflammatory factors [52, 53]. However, this hypothesis requires further validation. Gout not only exhibits circadian rhythm patterns but also displays seasonal variations, with flare-ups often occurring during the spring in the northern hemisphere [54]. This seasonal pattern may be linked to factors such as the yearly fluctuation of serum urate levels, changes in immune function throughout the year, and alterations in the gut microbiome [55]. This observation suggests that exploring rhythmic changes in autoimmune diseases may offer new directions for research. By studying these temporal variations, it is possible to enhance the understanding of disease mechanisms and improve clinical treatment approaches. Recognizing how fluctuations in factors like serum urate, immune function, and gut microbiome correspond to disease patterns can lead to more targeted and timely interventions, potentially leading to better patient outcomes and personalized care strategies. It is important to note that clinical recommendations should not rely solely on these temporal variables but should also incorporate a comprehensive assessment of the local socio-economic context [56].

The strengths of our study include the use of a fully transparent and effective assignment method to distinguish CircS, improving upon previously reported approaches. Additionally, to our knowledge, this is the first study to demonstrate associations of CircS with gout and hyperuricemia in a weighted, nationally representative sample of the U.S. population, suggesting a potential link between circadian rhythm disruption and these conditions. This also broadens the scope for exploring an important question: whether a common and central etiological feature underlies the clustering of MetS and CircS risk factors. However, several limitations should be acknowledged. First, the cross-sectional design limits the ability to establish causality in the observed associations. Second, the diagnosis of gout was based on self-reported data, which may introduce recall bias. Third, although multiple known confounders were adjusted for, the possibility of residual confounding from unmeasured variables cannot be ruled out. Finally, although the proportion of missing data was acceptable, it may still introduce potential bias.

To control the risk of gout and hyperuricemia, lifestyle changes are essential. This not only includes weight loss, increased physical activity, and dietary control, as recommended by current guidelines, but also addressing circadian misalignment. Improving sleep patterns and reducing shift work may offer a promising strategy for mitigating the risks of hyperuricemia and gout, ultimately leading to better health outcomes. Further exploration of circadian-controlled pathways in proteomics and metabolomics could lead to novel therapeutic strategies aimed at restoring circadian balance and improving health outcomes.

## Conclusion

In this nationally representative study, CircS was found to be significantly associated with both gout and hyperuricemia. A higher CircS score was linked to a progressively increased risk of these conditions. Elevated serum UA levels were also associated with a greater likelihood of CircS. However, due to the cross-sectional nature of this research, we were unable to infer causality directly. Prospective studies are warranted to confirm these associations and to explore the underlying mechanisms driving the relationship among CircS, gout, and hyperuricemia.

## Electronic supplementary material

Below is the link to the electronic supplementary material.

Supplementary Material 1

## Data Availability

No datasets were generated or analysed during the current study. The datasets used in this study are available in online repositories. These details can be accessed at: https://wwwn.cdc.gov/nchs/nhanes/, provided by the NCHS.
