# Supplementary material for: Associations of circadian syndrome with gout and hyperuricemia: a cross‑sectional analysis of NHANES 2007–2018
Source: BMC Public Health. 2025 Jun 10;25:2152. doi: 10.1186/s12889-025-23319-6 (PMC12150540; doi:10.1186/s12889-025-23319-6)
Supplement: Supplementary file 1 — Supplementary Material 1 [file 12889_2025_23319_MOESM1_ESM.pdf]

Variables	OR (95%CI)	P Value
UA	1.35(1.32,1.39)	<0.0001
Age	1.06(1.05,1.06)	<0.0001
PIR	0.93(0.91,0.95)	<0.0001
BMI	1.12(1.11,1.13)	<0.0001
Gender		
Female	Ref	
Male	1.02(0.96,1.08)	0.55
Race		
Mexican American	Ref	
Other Hispanic	1.00(0.88,1.14)	0.99
Non-Hispanic white	1.31(1.17,1.47)	<0.0001
Non-Hispanic black	1.13(0.99,1.29)	0.06
Other races	0.95(0.80,1.12)	0.54
Education		
Less than 9th grade	Ref	
9-11th grade	0.75(0.65,0.87)	<0.001
High school graduate	0.70(0.60,0.81)	<0.0001
Some college	0.61(0.53,0.70)	<0.0001
College graduate or above	0.39(0.34,0.45)	<0.0001
Marital		
Married	Ref	
Widowed	2.71(2.44,3.02)	<0.0001
Divorced	1.39(1.26,1.54)	<0.0001
Separated	0.99(0.82,1.18)	0.87
Never married	0.41(0.36,0.46)	<0.0001
Living with partner	0.58(0.50,0.67)	<0.0001
Smoke		
Never	Ref	
Former	1.87(1.69,2.06)	<0.0001
Now	1.14(1.04,1.26)	0.01
CKD		
No	Ref	
Yes	3.55(3.24,3.89)	<0.0001
Cancer		
No	Ref	
Yes	2.42(2.19,2.68)	<0.0001
CVD		
No	Ref	
Yes	6.77(6.05,7.59)	<0.0001
Stroke		
No	Ref	
Yes	4.80(4.12,5.59)	<0.0001
Alcohol		
Never	Ref	
Former	1.57(1.39,1.78)	<0.0001
Mild	0.88(0.78,0.99)	0.03
Moderate	0.57(0.48,0.67)	<0.0001
Heavy	0.52(0.46,0.59)	<0.0001
Missing	0.93(0.79,1.08)	0.33
Vigorous		
No	Ref	
Yes	0.77(0.71,0.84)	<0.0001
Moderate		
No	Ref	
Yes	0.84(0.78,0.91)	<0.0001
Gout		
No	Ref	
Yes	4.16(3.52,4.92)	<0.0001
Hyperuricemia		
No	Ref	
Yes	2.51(2.32,2.71)	<0.0001

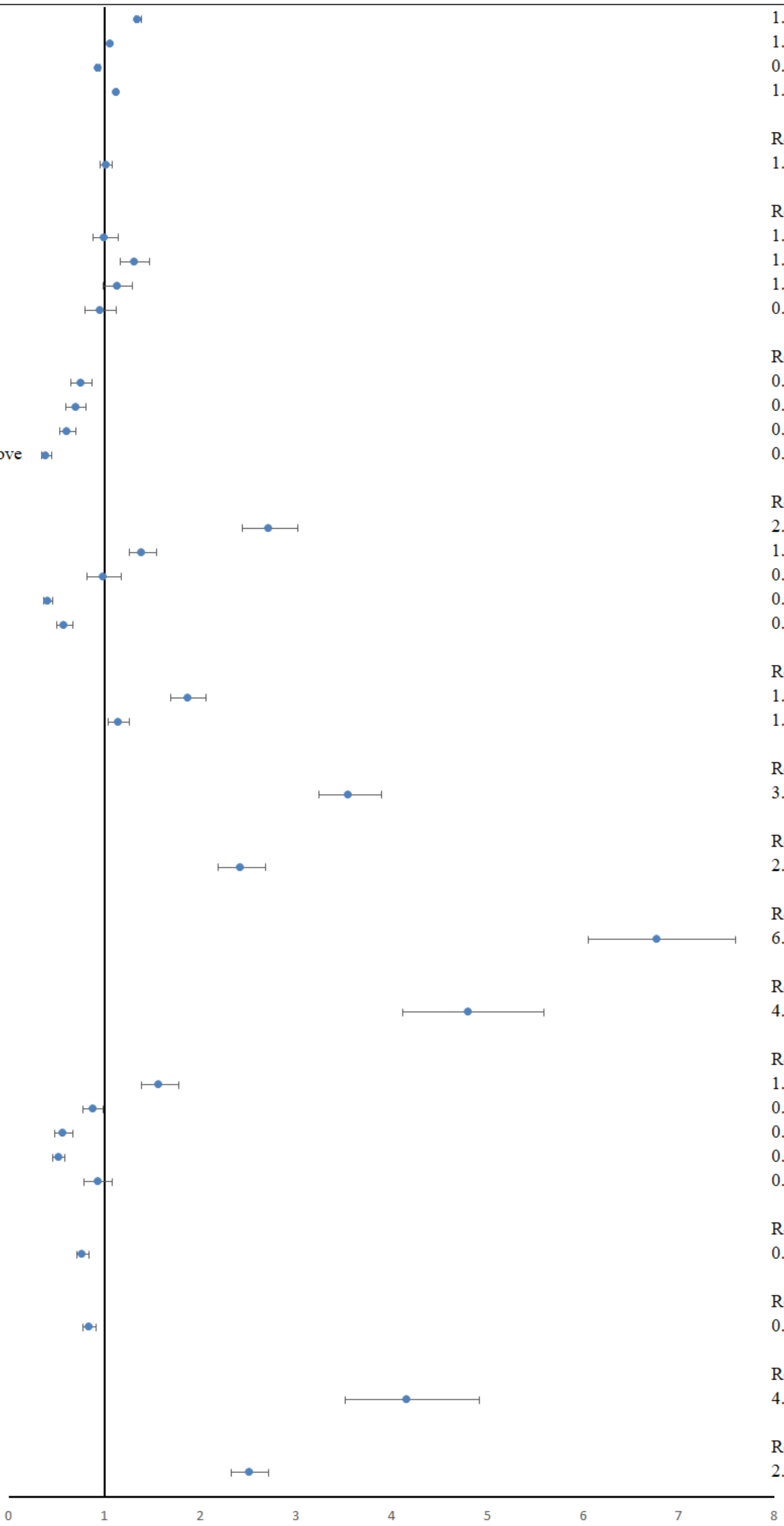

Figure S1 Logistic regression analysis of multiple risk factors and circadian syndrome.

CI, confidence interval; OR, odds ratio; PIR, poverty income ratio; BMI, body mass index; CKD, chronic kidney disease; CVD, cardiovascular disease; PHQ-9, Patient Health Questionnaire-9; DM, diabetes mellitus.

Variable	Total	Gout			Hyperuricemia		
		No (28748)	Yes (1409)	P Values	No (23988)	Yes (6169)	P Value
UA	5.41 (0.01)	5.37 (0.01)	6.50 (0.06)	< 0.0001	4.93 (0.01)	7.41 (0.02)	< 0.0001
Age	47.44 (0.23)	46.86 (0.23)	61.26 (0.49)	< 0.0001	46.56 (0.25)	51.08 (0.31)	< 0.0001
20-34	7527 (27.49)	7492 (99.54)	35 (0.46)		6363 (83.80)	1164 (16.20)	
35-49	7594 (27.58)	7416 (97.69)	178 (2.31)		6401 (84.18)	1193 (15.82)	
50-64	7929 (26.61)	7463 (94.19)	466 (5.81)		6156 (79.41)	1773 (20.59)	
>=65	7107 (18.32)	6377 (90.81)	730 (9.19)		5068 (72.42)	2039 (27.58)	
Gender				< 0.0001			< 0.0001
Female	15459 (51.57)	15039 (97.57)	420 (2.43)		12664 (83.56)	2795 (16.44)	
Male	14698 (48.43)	13709 (94.34)	989 (5.66)		11324 (77.56)	3374 (22.44)	
PIR	3.00 (0.04)	3.00 (0.04)	3.02 (0.07)	0.74	3.01 (0.04)	2.96 (0.04)	0.1
<=1.3	8848 (20.15)	8429 (95.96)	419 (4.04)		7059 (80.86)	1789 (19.14)	
1.3-3.5	10272 (32.78)	9784 (96.15)	488 (3.85)		8095 (79.56)	2177 (20.44)	
>3.5	8249 (39.58)	7864 (95.97)	385 (4.03)		6633 (81.52)	1616 (18.48)	
Missing	2788 (7.49)	2671 (95.69)	117 (4.31)		2201 (80.24)	587 (19.76)	
BMI	29.04 (0.09)	28.92 (0.08)	31.94 (0.31)	< 0.0001	28.21 (0.08)	32.54 (0.16)	< 0.0001
<25	8584 (29.37)	8367 (98.19)	217 (1.81)		7742 (91.63)	842 (8.37)	
25-30	9846 (32.82)	9425 (96.36)	421 (3.64)		8051 (82.59)	1795 (17.41)	
>=30	11450 (37.12)	10704 (94.04)	746 (5.96)		8002 (70.43)	3448 (29.57)	
Race				< 0.0001			< 0.0001
Mexican American	4604 (8.61)	4502 (98.32)	102 (1.68)		3906 (85.32)	698 (14.68)	
Other Hispanic	3190 (5.90)	3103 (97.89)	87 (2.11)		2691 (85.49)	499 (14.51)	
Non-Hispanic white	12480 (66.77)	11786 (95.59)	694 (4.41)		9789 (80.08)	2691 (19.92)	
Non-Hispanic black	6195 (10.65)	5835 (95.52)	360 (4.48)		4660 (77.54)	1535 (22.46)	
Other races	3688 (8.07)	3522 (96.22)	166 (3.78)		2942 (80.95)	746 (19.05)	
Education				0.25			< 0.0001
Less than 9th grade	3114 (5.25)	2950 (95.41)	164 (4.59)		2503 (80.94)	611 (19.06)	
9-11th grade	4202 (10.35)	4003 (96.05)	199 (3.95)		3340 (80.38)	862 (19.62)	
High school graduate	6866 (22.88)	6515 (95.69)	351 (4.31)		5387 (79.17)	1479 (20.83)	
Some college	8934 (31.58)	8527 (95.88)	407 (4.12)		6989 (78.85)	1945 (21.15)	
College graduate or above	7009 (29.88)	6721 (96.46)	288 (3.54)		5746 (83.73)	1263 (16.27)	
Marital				< 0.0001			< 0.0001
Married	15525 (55.39)	14682 (95.26)	843 (4.74)		12427 (81.15)	3098 (18.85)	
Widowed	2326 (5.59)	2143 (92.92)	183 (7.08)		1618 (70.29)	708 (29.71)	
Divorced	3321 (10.26)	3141 (95.58)	180 (4.42)		2587 (79.29)	734 (20.71)	
Separated	1020 (2.38)	971 (96.52)	49 (3.48)		829 (82.70)	191 (17.30)	
Never married	5501 (18.16)	5403 (98.59)	98 (1.41)		4481 (81.72)	1020 (18.28)	
Living with partner	2450 (8.19)	2394 (97.81)	56 (2.19)		2034 (83.06)	416 (16.94)	
Smoke				< 0.0001			< 0.0001
Never	16822 (55.69)	16236 (96.91)	586 (3.09)		13620 (81.79)	3202 (18.21)	
Former	7220 (24.71)	6626 (93.19)	594 (6.81)		5376 (76.42)	1844 (23.58)	
Now	6099 (19.56)	5870 (97.00)	229 (3.00)		4979 (82.75)	1120 (17.25)	
CKD				< 0.0001			< 0.0001

No	24546 (85.20)	23774 (97.01)	772 (2.99)		20432 (83.53)	4114 (16.47)	
Yes	5382 (14.16)	4761 (90.02)	621 (9.98)		3373 (63.27)	2009 (36.73)	
Cancer				< 0.0001			< 0.0001
No	27309 (89.85)	26178 (96.52)	1131 (3.48)		21879 (81.14)	5430 (18.86)	
Yes	2825 (10.06)	2548 (91.48)	277 (8.52)		2091 (76.36)	734 (23.64)	
CVD				< 0.0001			< 0.0001
No	26869 (91.38)	25939 (96.85)	930 (3.15)		21786 (81.70)	5083 (18.30)	
Yes	3286 (8.62)	2807 (87.03)	479 (12.97)		2201 (69.60)	1085 (30.40)	
Stroke				< 0.0001			< 0.0001
No	28950 (97.03)	27702 (96.24)	1248 (3.76)		23165 (80.96)	5785 (19.04)	
Yes	1175 (2.87)	1016 (88.22)	159 (11.78)		799 (70.32)	376 (29.68)	
PHQ-9				0.05			0.002
[0,9]	25589 (86.85)	24408 (96.00)	1181 (4.00)		20319 (80.59)	5270 (19.41)	
[10,27]	2592 (7.56)	2439 (95.35)	153 (4.65)		2010 (78.80)	582 (21.20)	
Missing	1976 (5.58)	1901 (97.06)	75 (2.94)		1659 (84.09)	317 (15.91)	
DM				< 0.0001			< 0.0001
No	21441 (75.99)	20774 (97.16)	667 (2.84)		17751 (83.29)	3690 (16.71)	
Pre-diabetes	2545 (8.43)	2399 (94.89)	146 (5.11)		1837 (71.57)	708 (28.43)	
Diabetes	5856 (14.50)	5262 (90.39)	594 (9.61)		4090 (70.81)	1766 (29.19)	
Hypertension				< 0.0001			< 0.0001
No	17372 (62.50)	17074 (98.40)	298 (1.60)		15028 (86.61)	2344 (13.39)	
Yes	12784 (37.50)	11673 (92.02)	1111 (7.98)		8959 (70.73)	3825 (29.27)	
Alcohol				< 0.0001			0.002
Never	3937 (10.01)	3796 (96.84)	141 (3.16)		3140 (80.67)	797 (19.33)	
Former	4277 (11.65)	3974 (93.47)	303 (6.53)		3286 (78.21)	991 (21.79)	
Mild	9242 (33.91)	8763 (95.55)	479 (4.45)		7391 (81.59)	1851 (18.41)	
Moderate	4257 (16.26)	4113 (97.29)	144 (2.71)		3438 (82.26)	819 (17.74)	
Heavy	5592 (19.89)	5396 (97.08)	196 (2.92)		4417 (78.78)	1175 (21.22)	
Missing	2852 (8.29)	2706 (95.32)	146 (4.68)		2316 (81.58)	536 (18.42)	
Vigorous				0.16			0.66
No	24199 (78.05)	23040 (95.89)	1159 (4.11)		19239 (80.73)	4960 (19.27)	
Yes	5951 (21.94)	5702 (96.41)	249 (3.59)		4744 (80.38)	1207 (19.62)	
Moderate				0.12			0.38
No	19038 (58.27)	18143 (96.19)	895 (3.81)		15110 (80.89)	3928 (19.11)	
Yes	11106 (41.71)	10593 (95.75)	513 (4.25)		8869 (80.33)	2237 (19.67)	
Short sleep				< 0.001			0.28
No	26008 (88.46)	24852 (96.20)	1156 (3.80)		20765 (80.77)	5243 (19.23)	
Yes	4074 (11.32)	3826 (94.63)	248 (5.37)		3162 (79.71)	912 (20.29)	
CircS				< 0.0001			< 0.0001
No	20773 (72.55)	20246 (97.77)	527 (2.23)		17590 (84.96)	3183 (15.04)	
Yes	9384 (27.45)	8502 (91.34)	882 (8.66)		6398 (69.26)	2986 (30.74)	
CircS scores				< 0.0001			< 0.0001
<=3	20773 (72.55)	20246 (97.77)	527 (2.23)		17590 (84.96)	3183 (15.04)	
4	5092 (15.28)	4708 (92.88)	384 (7.12)		3596 (71.15)	1496 (28.85)	

5	3453 (10.12)	3060 (89.31)	393 (10.69)		2270 (67.11)	1183 (32.89)
>=6	839 (2.04)	734 (89.89)	105 (10.11)		532 (65.71)	307 (34.29)

Table S1 Characteristics of participants by categories of gout and hyperuricemia: NHANES 2007–2018.

Data are presented as survey-weighted mean (stand error) for continuous variables and sample (survey-weighted percentage) for categorical variables. The t-test for slope was used in survey-weighted generalized linear models. UA, uric acid; PIR, poverty income ratio; BMI, body mass index; CKD, chronic kidney disease; CVD, cardiovascular disease; PHQ-9, Patient Health Questionnaire-9; DM, diabetes mellitus; CircS, circadian syndrome.

	Gout			Hyperuricemia		
Variables	No	Yes	P value	No	Yes	P value
Age						
20-34	Ref	10.04(4.20,23.99)	<0.0001	Ref	3.60(2.91,4.45)	<0.0001
35-49	Ref	3.94(2.79,5.58)	<0.0001	Ref	2.48(2.05,3.00)	<0.0001
50-64	Ref	2.25(1.64,3.09)	<0.0001	Ref	2.14(1.84,2.49)	<0.0001
>=65	Ref	1.95(1.59,2.39)	<0.0001	Ref	1.94(1.62,2.31)	<0.0001
Gender						
Female	Ref	4.53(3.43,5.99)	<0.0001	Ref	4.00(3.56,4.48)	<0.0001
Male	Ref	4.04(3.35,4.88)	<0.0001	Ref	1.68(1.49,1.89)	<0.0001
PIR						
<=1.3	Ref	4.67(3.58,6.08)	<0.0001	Ref	2.79(2.40,3.26)	<0.0001
1.3-3.5	Ref	4.19(3.17,5.52)	<0.0001	Ref	2.59(2.24,2.99)	<0.0001
>3.5	Ref	3.96(3.12,5.03)	<0.0001	Ref	2.23(1.93,2.58)	<0.0001
Missing	Ref	4.29(2.51,7.33)	<0.0001	Ref	2.77(2.12,3.63)	<0.0001
BMI						
<25	Ref	4.34(2.52,7.47)	<0.0001	Ref	2.86(2.13,3.85)	<0.0001
25-30	Ref	3.07(2.38,3.95)	<0.0001	Ref	1.99(1.72,2.30)	<0.0001
>=30	Ref	3.54(2.78,4.50)	<0.0001	Ref	1.54(1.37,1.72)	<0.0001
Race						
Mexican American	Ref	3.94(2.16,7.19)	<0.0001	Ref	1.90(1.56,2.32)	<0.0001
Other Hispanic	Ref	5.01(2.88,8.72)	<0.0001	Ref	2.98(2.35,3.78)	<0.0001
Non-Hispanic white	Ref	3.90(3.17,4.80)	<0.0001	Ref	2.49(2.25,2.77)	<0.0001
Non-Hispanic black	Ref	5.41(3.92,7.45)	<0.0001	Ref	3.01(2.68,3.37)	<0.0001
Other races	Ref	4.06(2.51,6.56)	<0.0001	Ref	2.18(1.67,2.83)	<0.0001
Education						
Less than 9th grade	Ref	4.56(2.79,7.46)	<0.0001	Ref	2.86(2.26,3.61)	<0.0001
9-11th grade	Ref	3.34(2.30,4.85)	<0.0001	Ref	2.66(2.23,3.16)	<0.0001
High school graduate	Ref	3.45(2.48,4.81)	<0.0001	Ref	1.97(1.66,2.34)	<0.0001
Some college	Ref	4.62(3.36,6.36)	<0.0001	Ref	2.72(2.39,3.09)	<0.0001
College graduate or above	Ref	4.68(3.46,6.35)	<0.0001	Ref	2.60(2.17,3.11)	<0.0001
Marital						
Married	Ref	4.06(3.29,5.01)	<0.0001	Ref	2.49(2.25,2.77)	<0.0001
Widowed	Ref	2.36(1.41,3.95)	0.001	Ref	2.13(1.67,2.72)	<0.0001
Divorced	Ref	2.06(1.30,3.28)	0.003	Ref	2.45(1.93,3.12)	<0.0001
Separated	Ref	4.20(2.04,8.65)	<0.001	Ref	2.05(1.36,3.10)	<0.001
Never married	Ref	5.05(2.95,8.66)	<0.0001	Ref	2.57(2.03,3.24)	<0.0001
Living with partner	Ref	6.43(3.53,11.71)	<0.0001	Ref	2.64(1.99,3.51)	<0.0001
Smoke						
Never	Ref	5.21(4.13,6.56)	<0.0001	Ref	2.74(2.45,3.07)	<0.0001
Former	Ref	2.98(2.21,4.02)	<0.0001	Ref	2.22(1.85,2.66)	<0.0001
Now	Ref	3.09(2.10,4.56)	<0.0001	Ref	2.10(1.76,2.49)	<0.0001
CKD						

No	Ref	4.18(3.36,5.20)	<0.0001	Ref	2.18(2.00,2.37)	<0.0001
Yes	Ref	2.07(1.61,2.68)	<0.0001	Ref	2.07(1.82,2.36)	<0.0001
Cancer						
No	Ref	4.23(3.51,5.10)	<0.0001	Ref	2.51(2.30,2.75)	<0.0001
Yes	Ref	2.59(1.78,3.76)	<0.0001	Ref	2.30(1.86,2.84)	<0.0001
CVD						
No	Ref	3.89(3.24,4.66)	<0.0001	Ref	2.47(2.27,2.69)	<0.0001
Yes	Ref	1.55(1.17,2.05)	0.002	Ref	1.70(1.32,2.19)	<0.0001
Stroke						
No	Ref	4.10(3.47,4.85)	<0.0001	Ref	2.48(2.29,2.68)	<0.0001
Yes	Ref	2.10(1.11,3.95)	0.02	Ref	2.24(1.52,3.32)	<0.0001
DM						
No	Ref	3.67(2.88,4.67)	<0.0001	Ref	2.29(2.08,2.53)	<0.0001
Pre-diabetes	Ref	2.42(1.47,3.98)	<0.001	Ref	2.07(1.64,2.61)	<0.0001
Diabetes	Ref	2.28(1.62,3.21)	<0.0001	Ref	1.81(1.49,2.18)	<0.0001
Hypertension						
No	Ref	3.60(2.61,4.97)	<0.0001	Ref	2.26(1.96,2.60)	<0.0001
Yes	Ref	2.07(1.63,2.61)	<0.0001	Ref	1.56(1.41,1.73)	<0.0001
Alcohol						
Never	Ref	2.97(1.85,4.75)	<0.0001	Ref	3.07(2.53,3.71)	<0.0001
Former	Ref	3.26(2.27,4.68)	<0.0001	Ref	2.87(2.37,3.47)	<0.0001
Mild	Ref	3.95(2.96,5.26)	<0.0001	Ref	2.54(2.19,2.94)	<0.0001
Moderate	Ref	5.03(2.89,8.75)	<0.0001	Ref	2.50(1.98,3.16)	<0.0001
Heavy	Ref	4.90(3.21,7.48)	<0.0001	Ref	1.92(1.60,2.30)	<0.0001
Missing	Ref	3.62(2.05,6.41)	<0.0001	Ref	3.38(2.41,4.73)	<0.0001
Vigorous						
No	Ref	4.04(3.32,4.92)	<0.0001	Ref	2.69(2.45,2.96)	<0.0001
Yes	Ref	4.62(3.24,6.58)	<0.0001	Ref	1.94(1.61,2.33)	<0.0001
Moderate						
No	Ref	4.59(3.63,5.79)	<0.0001	Ref	2.84(2.58,3.12)	<0.0001
Yes	Ref	3.76(2.93,4.82)	<0.0001	Ref	2.11(1.86,2.41)	<0.0001

Table S2 Stratified analysis for the association of circadian syndrome with both gout and hyperuricemia.

Data are presented as survey-weighted mean (stand error) for continuous variables and sample (survey-weighted percentage) for categorical variables. The t-test for slope was used in survey-weighted generalized linear models. UA, uric acid; PIR, poverty income ratio; BMI, body mass index; CKD, chronic kidney disease; CVD, cardiovascular disease; PHQ-9, Patient Health Questionnaire-9; DM, diabetes mellitus.

	Gout						Hyperuricemia					
	Crude model		Model 1		Model 2		Crude model		Model 1		Model 2	
CircS scores	OR (95%CI)	P value	OR (95%CI)	P value	OR (95%CI)	P value	OR (95%CI)	P value	OR (95%CI)	P value	OR (95%CI)	P value
0	ref		ref		ref		ref		ref		ref	
1	2.34(1.20, 4.58)	0.01	1.38(0.68,2.77)	0.36	1.53(0.76,3.07)	0.22	1.81(1.49, 2.21)	<0.0001	1.36(1.11,1.66)	0.003	1.45(1.18,1.80)	<0.001
2	5.00(2.64, 9.48)	<0.0001	1.98(0.99,3.97)	0.05	2.16(1.07,4.36)	0.03	2.85(2.41, 3.38)	<0.0001	1.60(1.31,1.94)	<0.0001	1.74(1.41,2.16)	<0.0001
3	8.14(4.63,14.32)	<0.0001	2.29(1.23,4.26)	0.01	2.57(1.30,5.10)	0.01	4.31(3.62, 5.14)	<0.0001	1.90(1.57,2.31)	<0.0001	2.16(1.75,2.68)	<0.0001
4	13.98(7.84,24.93)	<0.0001	2.62(1.31,5.21)	0.01	2.89(1.43,5.81)	0.004	5.60(4.75, 6.61)	<0.0001	2.05(1.68,2.50)	<0.0001	2.33(1.86,2.92)	<0.0001
5	21.83(11.79,40.43)	<0.0001	2.85(1.34,6.07)	0.01	3.24(1.49,7.04)	0.004	6.77(5.71, 8.04)	<0.0001	2.01(1.63,2.48)	<0.0001	2.45(1.95,3.10)	<0.0001
6	20.67(10.80,39.57)	<0.0001	2.38(1.04,5.48)	0.04	2.61(1.12,6.12)	0.03	7.12(5.30, 9.56)	<0.0001	1.86(1.37,2.54)	<0.001	2.69(1.79,4.05)	<0.0001
7	19.12(7.92,46.16)	<0.0001	1.84(0.63,5.43)	0.26	2.21(0.71,6.83)	0.17	8.02(4.31,14.90)	<0.0001	1.87(1.03,3.37)	0.04	3.77(1.96,7.24)	<0.001
p for trend		**<0.0001**		**0.001**		**0.001**		**<0.0001**		**<0.0001**		**<0.0001**

Table S3 Multivariate regression analysis and trend text of the association of circadian scores with both gout and hyperuricemia.
Crude model: Circadian scores
Model 1 for gout: Circadian scores, Age, BMI, Gender, Cancer, CVD, Stroke, CKD, DM, Hypertension, Short sleep
Model 1 for Hyperuricemia: Circadian scores, Age, BMI, Gender, Cancer, CVD, Stroke, CKD, DM, Hypertension
Model 2: Circadian scores, Age, PIR, BMI, Gender, Race, Education, Marital, Smoke, Cancer, CVD, CKD, Stroke, PHQ-9, DM, Hypertension, Alcohol, Vigorous, Moderate, Short sleep
CI, confidence interval; OR, odds ratio; PIR, poverty income ratio; BMI, body mass index; CKD, chronic kidney disease; CVD, cardiovascular disease; PHQ-9, Patient Health Questionnaire-9; DM, diabetes mellitus.
